# The complete chloroplast genome of *Aquilegia barnebyi*, a basal eudicot species

**DOI:** 10.1080/23802359.2020.1719919

**Published:** 2020-02-06

**Authors:** Hui Huang

**Affiliations:** Kunming Institute of Botany, Chinese Academy of Science, Kunming, China

**Keywords:** *Aquilegia barnebyi*, basal eudicot species, chloroplast genome, phyologenetic analyze

## Abstract

*Aquilegia barnebyi,* belonging to the genus *Aquilegia* (Ranunculaceae), is a member of basal eudicot species. In this study, we obtained the complete chloroplast (cp) genome of *A. barnebyi*. The genome size is 161,954 bp with a GC content of 38.98%. A total of 113 unique genes including 79 protein-coding genes, 30 tRNA genes, four rRNA genes were annotated. The large single-copy region and small single-copy region contains 91,250 bp and 17,359 bp, respectively. The inverted repeat regions are 26,671 bp in length. The phyologenetic analysis indicated that *A. barnebyi* had a close relationship with *A. coerulea*. And four species in genus *Aquilegia* formed a monophyletic group with high support value. The availability of *A. barnebyi* cp genomic resources will greatly helpful for taxonomy, phylogeny and conservation genetic studies of basal eudicot specie.

The *Aquilegia* genus (Ranunculaceae) belongs to basal eudicot angiosperms which consists of approximately 75 species occurring in North America (22 spp), Asia (23 spp), and Europe (21 spp) (Nold 2003). These species have a number of characteristics that show their primitive evolution (Zahn et al. 2010). The investigation of the *Aquilegia* genus can serve as an evolutionary link between core eudicots and monocots. Furthermore, as a classic example of adaptive radiation, the *Aquilegia* genus has outstanding potential as a subject of evolutionary studies (Kramer and Hodges 2010). However, the little genetic and genomic information of the *Aquilegia* has hindered the study of the genus. In this study, we report the cp genome of *A. barnebyi,* thus providing useful information for taxonomy, evolutionary dynamics and conservation studies of the *Aquilegia* genus and basal eudicot angiosperms.

The leaves *of A. barnebyi* were collected from rifle falls of northwest Colorado, USA (39°40′35″N, 107°41′57″W), and the speciman was deposited at the herbarium of department of ecology, evolution and marine biology, university of California (speciman code Aquilegia_BA). The chloroplast reads were filtered from whole genome Illumina sequencing data of *A. barnebyi* which was downloaded from NCBI Short Read Archive (SRR7965809) (Filiault et al. 2018). We mapped all the sequencing reads to the *A. rockii* cp genome using bowtie2 (v2.3.4.3) (Langmead et al. 2009; Yu et al. 2019). The mapped chloroplast reads were assembled using Geneious v7.1.7 (Biomatters, New Zealand) with the *A. rockii* cp genome as reference. The complete cp genome was annotated using the program DOGMA (Wyman et al. 2004).Circular genome map was drawn using OGDRAW (Lohse et al. 2013).

The complete cp genome of *A. barnebyi* was composed of single circular double-stranded DNA molecules and was 161,954 bp in length. The data was deposited in GenBank with the accession number MN882557. As other taxa in the family of Ranunculaceae (Yu et al. 2019), the cp genome of *A. barnebyi* displayed the typical quadripartite structure, including a pair of inverted repeat regions (IR with 26,671 bp) divided by two single-copy regions (LSC 91,250 bp and SSC 17,359 bp). The overall GC content of the cp genome was 38.98%. There were a total of 113 unique genes, including 79 protein-coding genes, 30 tRNA genes and 4 rRNA genes, in *A. barnebyi* cp genome. Among these genes, nine protein-coding genes and six tRNA genes contained a single intron, and two protein-coding genes possessed two introns. The gene *rps12* found to be trans-spliced; with the 5′-end exon located in the LSC region and two copies of 3′-end exon and intron in the IR regions. Moreover, the *rpl32*, *infA* and *clpP* were identified as pseudogenes because of the partial duplication.

To understand the phylogenetic position of *A. barnebyi* within the genus *Aquilegia* and the family Ranunculaceae, we downloaded the complete cp genome of nine species in Ranunculaceae including three species in genus *Aquilegia.* The sequences were aligned using MAFFT v7.307 (Katoh and Standley 2013), and RAxML (Stamatakis 2014) was used to construct a maximum likelihood tree with *Ranunculus occidentalis* as outgroup. All nodes in the complete plastome tree were strongly supported. The phylogenetic tree showed that four species in genus *Aquilegia* formed a monophyletic group with high support value. And *A. barnebyi* was closely related to *A. coerulea* and far away from *A. rockii* (Figure 1). This published *A. barnebyi* cp genome will provide useful information for phyogenetic and evolutionary studies in the genus *Aquilegia* and Ranunculaceae.

**Figure 1.**
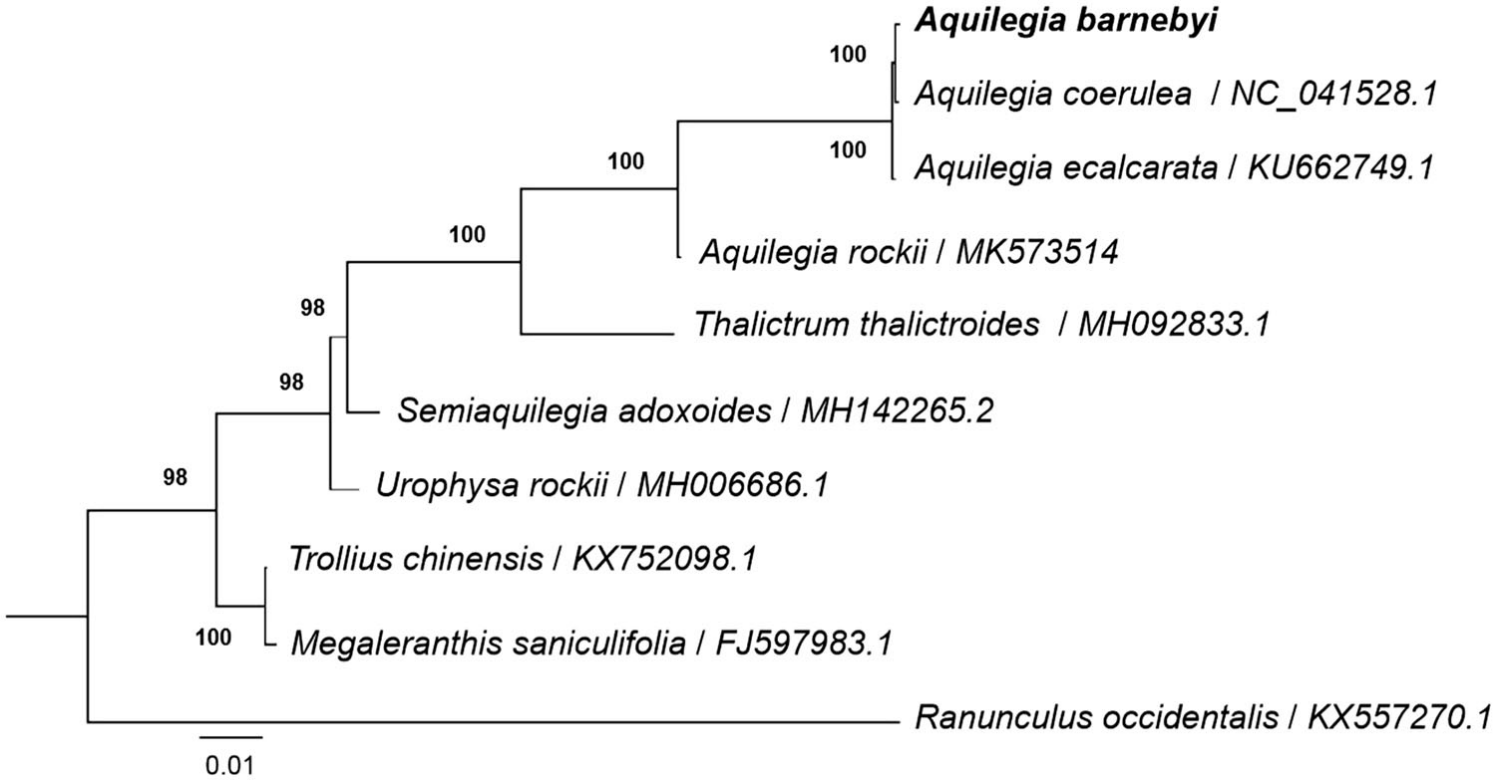
Phylogenetic tree reconstruction of 10 taxa in Ranunculaceae including 4 taxa in *Aquilegia* using maximum likelihood (ML) methods based on whole cp genomes. ML bootstrap support value presented at each node.
